# Potential therapeutic options for COVID-19: an update on current evidence

**DOI:** 10.1186/s40001-021-00626-3

**Published:** 2022-01-13

**Authors:** Zahra Niknam, Ameneh Jafari, Ali Golchin, Fahima Danesh Pouya, Mohadeseh Nemati, Mostafa Rezaei-Tavirani, Yousef Rasmi

**Affiliations:** 1 Proteomics Research Center, Shahid Beheshti University of Medical Sciences, Tehran, Iran; 2 Advanced Therapy Medicinal Product (ATMP) Department, Breast Cancer Research Center, Motamed Cancer Institute, ACECR, Tehran, Iran; 3 Department of Clinical Biochemistry and Applied Cell Sciences, School of Medicine, Urmia University of Medical Sciences, Urmia, Iran; 4 Department of Biochemistry, School of Medicine, Urmia University of Medical Sciences, Urmia, Iran; 5 Proteomics Research Center, Faculty of Paramedical Sciences, Shahid Beheshti University of Medical Sciences, Tehran, Iran; 6 Cellular and Molecular Research Center, Urmia University of Medical Sciences, Urmia, Iran

**Keywords:** COVID-19, Repurposed drugs, Convalescent plasma, Monoclonal antibodies, Immunoglobulins, Cell therapy

## Abstract

SARS-CoV-2, a novel coronavirus, is the agent responsible for the COVID-19 pandemic and is a major public health concern nowadays. The rapid and global spread of this coronavirus leads to an increase in hospitalizations and thousands of deaths in many countries. To date, great efforts have been made worldwide for the efficient management of this crisis, but there is still no effective and specific treatment for COVID-19. The primary therapies to treat the disease are antivirals, anti-inflammatories and respiratory therapy. In addition, antibody therapies currently have been a many active and essential part of SARS-CoV-2 infection treatment. Ongoing trials are proposed different therapeutic options including various drugs, convalescent plasma therapy, monoclonal antibodies, immunoglobulin therapy, and cell therapy. The present study summarized current evidence of these therapeutic approaches to assess their efficacy and safety for COVID-19 treatment. We tried to provide comprehensive information about the available potential therapeutic approaches against COVID-19 to support researchers and physicians in any current and future progress in treating COVID-19 patients.

## Introduction

Coronaviruses, a large family of viruses, has been divided into four genera: α, β, γ, and δ. These viruses can lead to infection and illness in both animals and humans. Seven coronaviruses can infect humans worldwide, but four of them, such as 229E, NL63, OC43, and HKU1, commonly infect individuals [1, 2]. They generally lead to diseases ranging from the common cold to severe infectious respiratory illnesses, such as the most recently appeared coronavirus disease 2019 (COVID-19), leads to thousands of deaths in many countries around the world [3]. Patients infected with SARS-CoV-2 generally have major clinical symptoms and signs, such as fever, dyspnea and cough [4, 5]. In addition, minor symptoms, such as dysgeusia, anosmia, gastrointestinal symptoms, headache and skin lesions were increasingly reported in COVID-19 patients [4–6]. This novel coronavirus, which belongs to the β coronaviruses family, can lead to serious disorders, such as chronic lung disease, acute respiratory distress syndrome (ARDS), cardiac and renal injury, especially in patients with older age and comorbidities (diabetes mellitus, hypertension, and heart failure) [7]. Severe acute respiratory syndrome coronavirus-2 (SARS-CoV-2) begins its infection through the priming of spike (S) protein by transmembrane protease serine 2 (TMPRSS2) on the host cell membrane and interaction of S protein with angiotensin-converting enzyme 2 (ACE2) receptors [8, 9]. The virus-cell interactions and the rapid viral replications stimulate the production of various pro-inflammatory cytokines and chemokines, lead to immunopathogenesis [10, 11].

In general, SARS-CoV-2-infected persons can be divided into three groups: (I) asymptomatic cases with or without detectable virus, (II) non-severe or mild symptomatic cases with the presence of the virus that usually recover, and (III) severe respiratory symptomatic patients with more viral load, multi-organ failure, and primarily respiratory failure that require hospitalization [7, 12]. Based on the evidence, most COVID-19 patients have a mild or moderate infection, but up to 5–10% have a sever and even life-threatening disease course [13]. Therefore, it is necessary to find strategies for control of SARS-CoV-2. In this regard, the COVID-19 vaccine program continues to be implemented globally; however, effective treatments still need to be identified, especially in countries, where vaccination is slow, and mutations can potentially threaten vaccine escape. In addition, some people may not respond fully to COVID-19 vaccines, such as patients on chemotherapy, patients with hematologic malignancies, immunocompromised people, and others. From the onset of COVID-19 until now, various studies and clinical trials have been conducted to develop a treatment against the disease, but most are yet in the early stages of research.

In this review, the current evidence of potential therapeutic options for COVID-19, including antivirals, anti-inflammatories, convalescent plasma therapy, monoclonal antibodies, immunoglobulin therapy, cell therapy, and nutraceutical supplementation will be discussed. Our aim is to assist scientists and physicians engaged in this ongoing pandemic to improve and develop their plans for further research and therapeutic management.

## Treatment strategies for COVID-19

### Potentially recommended drugs

Nowadays, due to the urgent need to overcome the SARS-CoV-2 pandemic, extensive global researches are underway to find appropriate drug agents to treat COVID-19. As developing a novel drug is a lengthy, expensive and risky process, to discover a cure for this pandemic, the emergency of the pandemic considered to first attempts to identify possible treatments between approved or marketed drugs. Drug repurposing is the best approach to recognize therapeutic options for COVID‑19 in a limited time [14, 15]. The great benefit of this approach is the available science about the metabolic profile of drugs, dosages requirement, possible risks, complications and side effects. The drawback of these classes of medicines is their inadequate selectivity and, mostly, weak utility among families of viruses [15].

Various drugs applied in interventional clinical trials launched by the World Health Organization (WHO) can be classified according to their nature and complementary effect. Therefore, antivirals, anti-inflammatory, anticoagulants, and some common drugs together with combination therapies are used on ongoing investigations for inhibition, treatment, or supportive care. Here we discussed an updated list of the most commonly used drugs in COVID-19 treatment.

Remdesivir is the first drug that recently received Food and Drug Administration (FDA) emergency use authorization to treat SARS-CoV-2. Remdesivir, an RNA-dependent RNA polymerase (RdRp) inhibitor, has shown inhibitory effects on SARS-CoV-2, both in vitro and in vivo [16]. Nowadays, remdesivir has been widely used, and many clinical trials are underway to evaluate its efficacy on COVID-19. In some studies, an improved clinical result was observed, but also multiple adverse effects in the remdesivir-treated group were occurred [17, 18]. Moreover, contrasting results have been reported in different nations owing to the small number of randomized clinical trials, varied study designs, genetic reasons and different treatment regimens (5 or 10 days) [19]. The recommended regiment for remdesivir is 200 mg IV once, then remdesivir 100 mg IV once daily for 4 days. However, if there is no substantial clinical improvement by day 5, treatment may be extended to 10 days. In patients with moderate COVID-19 who do not require supplemental oxygen, there is insufficient evidence to recommend either for or against the routine use of remdesivir, but use may be appropriate in patients at high risk of disease progression. Some meta-analysis of clinical trials concluded that remdesivir has shown in little to no mortality benefit, but probably improves the clinical output and decreases serious adverse events of hospitalized patients with COVID-19. In addition, it may lead to a small reduction in the proportion receiving ventilation and higher rates of hospital discharge [19–21]. Based on CDC’s guideline, in hospitalized patients who need supplemental oxygen (but without any device and ventilator), remdesivir or dexamethasone plus remdesivir is recommended. One recent clinical study has revealed that combination therapy of remdesivir plus baricitinib as a Janus kinase inhibitor, has superior effects compared to remdesivir alone in decreasing recovery time and accelerating improvement in clinical status between patients with COVID-19, considerably between those receiving high-flow oxygen or noninvasive ventilation [22]. In addition, fewer serious adverse events were observed in this combination therapy. Then in November 2021, the FDA issued an emergency use authorization for the baricitinib/remdesivir combination therapy for treatment on hospitalized COVID-19 patients who require supplemental oxygen.

Baricitinib on July 29, 2021, received FDA’s emergency use authorization for apply in hospitalized patients who require supplemental oxygen. Baricitinib is a suppressor of the JAK-STAT pathway, which is used for preventing pro-inflammatory cytokine release and systemic inflammation in interferonopathies. Baricitinib also is a numb-associated kinase (NAK) inhibitor, with an especially high affinity for AP2-associated protein kinase 1 (AAK1) a main member of the NAK family. AAK1 binds to clathrin and phosphorylates the medium subunit of AP2 (adaptor protein 2), playing an essential role in regulating clathrin‑mediated endocytosis and its suppression was indicated to decrease the infectivity of a large number of viruses. An artificial intelligence method identified the NAK family members as potential therapeutic targets against SARS‑CoV‑2 [23, 24]. Therefore, baricitinib may have both antiviral and anti-inflammatory properties in COVID-19. According to the new National Institutes of Health (NIH) guidelines, using baricitinib in combination with remdesivir was recommended. However, blockage of JAK-STAT pathway leads to inhibition of IFN production which may cause impairment of anti-viral immunity [25]. Currently, 20 clinical trials are ongoing for evaluating baricitinib effect on COVID-19. In hospitalized patients who have rapidly increasing oxygen needs, require high-flow oxygen or noninvasive ventilation, and have increased markers of inflammation, adding either baricitinib or IV tocilizumab to one of two therapeutic options (dexamethasone or dexamethasone plus remdesivir) is recommended. A meta-analysis of clinical trials involving a total of 2367 participants investigated the safety and efficacy of JAK-inhibitors including baricitinib and ruxolitinib in COVID-19 patients [26]. The results showed that JAK-inhibitors reduced needing invasive mechanical ventilation and had a borderline effect on rates of transferring to ICU and on ARDS. JAK-inhibitors did not reduce the time of hospitalization. The duration of JAK-inhibitor treatment of COVID-19 may be the main factor in determining its efficacy [26].

Dexamethasone is a corticosteroid used as an anti-inflammatory drug and is very promising in the treatment of COVID-19. Dexamethasone leads to a decrease in mortality rates, especially in seriously ill patients (on ventilator support or needing oxygen therapy). One study entered 2104 patients who received a dose of 6 mg per day for 10 days compared to the standard of care in 4321 patients. The risk of death was decreased by one-third in the dexamethasone arm in ventilated patients [27]. The results of the several clinical trials led to the wide application of dexamethasone for patients on a ventilator or receiving supplemental oxygen. According to literatures, corticosteroids were associated with reduced all-cause mortality [28, 29]. Using corticosteroids in severe COVID-19 patients reduced the occurrence of composite disease progression, but not enhanced the incidence of serious adverse events [28]. According to CDC recommendations, the dosing regimen for dexamethasone is 6 mg IV or oral once daily for up to 10 days or until hospital discharge. Moreover, if dexamethasone is not available, alternative glucocorticoids such as prednisone (40 mg), methylprednisolone (32 mg), or hydrocortisone (160 mg) can be used.

Heparin as an anticoagulant agent has been used in COVID-19 patients from prophylactic to therapeutic doses for preventing the formation of blood clots [30]. During SARS-CoV-2 infection, the alveolar damage and the pulmonary microvascular thrombosis due to the direct viral infection of endothelial cells are the major causes of acute lung injury [30, 31]. Hence, COVID-19-associated coagulopathy is occurred due to activation of the immune system and thromboinflammatory responses and endotheliopathy [31]. Histopathological studies demonstrated that endotheliitis with diffuse endothelial inflammation and microvascular and macrovascular thrombosis occur in the venous and arterial circulations leading to multiorgan dysfunction and thrombotic complications in COVID-19 [30, 31]. A recent randomized controlled trial indicated that the use of therapeutic-dose anticoagulation with heparin compared to usual-care thromboprophylaxis in noncritically ill COVID-19 patients enhanced the probability of survival to hospital discharge with decreased use of cardiovascular or respiratory organ support [32]. For COVID-19 patients admitted to the ICU, the results of another randomized trial do not support the routine empirical use of intermediate-dose prophylactic anticoagulation [33]. In a retrospective study, early heparin treatment indicated lower mortality risk in patients with COVID-19 [4]. According to the last update of COVID-19 treatment guidelines (February 11, 2021) (https://www.covid19treatmentguidelines.nih.gov/), anticoagulants and antiplatelet therapy should not be used for nonhospitalized COVID-19 patients. However, nonpregnant hospitalized adults with COVID-19 should receive prophylactic dose anticoagulation. At present, there is inadequate data to recommend either for or against the use of thrombolytics or higher than the prophylactic dose of anticoagulation for the prevention of venous thromboembolism in hospitalized COVID-19 patients.

Some repurposed drugs under evaluation in clinical trials for the treatment of COVID-19 are described in Table 1.

**Table 1:** Summarizes of potential drugs under investigation in clinical trials against COVID-19

Drugs	Drug target	Related disease	Results of studies	References
Antivirals				
Favipiravir	RdRp inhibitor	Influenza	Clinical improvement and viral clearance within 7 or 14 days, lower needing to supplemental oxygen therapy	[34–36]
Sofosbuvir/daclatasvir	Nucleoside analog/NS5A inhibitor	HCV	Improving clinical outcomes, reduce mortality rate and need for ICU/IMV	[37, 38]
Molnupiravir	RNA mutagenesis	Influenza	Highly effective at reducing nasopharyngeal SARS-CoV-2 infectious virus and viral RNA, has a favorable safety and tolerability profile	[39]
Danoprevir	NS3/4A protease inhibitor	HCV	Significantly shorter mean time to achieve both negative nucleic acid testing and hospital stays	[40]
Anti-inflammatory drugs				
Ruxolitinib	JAK inhibition	Rheumatoid arthritis	Faster clinical improvement, significant chest CT improvement	[41, 42]
Tofacitinib	JAK inhibition	Rheumatoid arthritis	Lower risk of death or respiratory failure through day 28	[43]
Imatinib	JAK inhibition	Cancer	Beneficial effects on survival and duration of mechanical ventilation	[44]
Fluvoxamine	Agonist for the sigma-1 receptor	Anti-depressant	Lower likelihood of clinical deterioration over 15 days	[45]
Methylprednisolone	Inhibition of proinflammatory cytokine production	Inflammation, immune system disorders	Decreased the recovery time, the need for transfer to intensive care and the severity markers C-reactive protein, D-dimer and LDH, lower need for a ventilator	[46–48]
Budesonide	Inhibition of proinflammatory cytokine production	Asthma	Reduced the likelihood of needing urgent medical care and reduced time to recovery, reduced hospital admissions or deaths	[49, 50]
Artesunate	NF-κB-coronavirus effect and chloroquine-like endocytosis inhibition	Malaria	Lower treatment time, improve prognosis and eliminate pathogens, with fewer adverse reactions	[51]
Type I interferons	Balances the expression of pro- and anti-inflammatory agents	Multiple sclerosis	Decreased mortality rate and time of hospitalization	[52, 53]
Other most common drugs				
Telmisartan	Angiotensin receptor blocker	Hypertension	Safe and reduced morbidity and mortality in hospitalized patients, anti-inflammatory effects	[54, 55]
Nitazoxanide	Inhibition of the pyruvate: ferredoxin/flavodoxin oxidoreductase cycle	Anti-parasitic	Improvement in clinical, virologic and inflammatory outcomes in moderate COVID-19, safe and significantly reduced viral load in early use	[56, 57]
Niclosamide	Prevention of viral entry by altering endosomal pH, Prevention of viral replication by inhibition of autophagy	Anti-parasitic	Accelerated time to recovery about 3 to 5 days in moderate to severe COVID-19 patients especially those with co-morbidities	[58]
Bromhexine	TMPRSS2 protease blocker	Mucolytic	The early administration reduced the ICU transfer, intubation, and the mortality rate	[59, 60]
Dornase alfa	Recombinant human deoxyribonuclease I	Cystic fibrosis	Improvement in oxygenation, reduction in ventilatory support	[61, 62]
Dexmedetomidine	Selective alpha-2 adrenoceptor agonist	Sedation	Effective sedative and may improve oxygenation, significantly reduced the intubation rate and ICU length of stay by 2.9 days, did not change the mortality rate decline in heart rate and high incidence of bradycardia and hypotension	[63, 64]
Fluoxetine	Selective serotonin reuptake inhibitor	Antidepressant	Lower risk of death or intubation in hospitalized patients	[65]

### Antibody-based immunotherapeutic strategies

Based on exiting evidence and previous experience in treating other viral infections, antibody‐based treatments as a kind of passive immunotherapy may be appropriate to achieve COVID-19 treatment [66]. In this section, immunotherapy based on the antibody include neutralizing monoclonal antibodies, and intravenous immunoglobulins as effective strategies for clinical treatment of SARS-CoV-2 infection have been discussed.

#### Convalescent plasma therapy

**Fig. 1:**
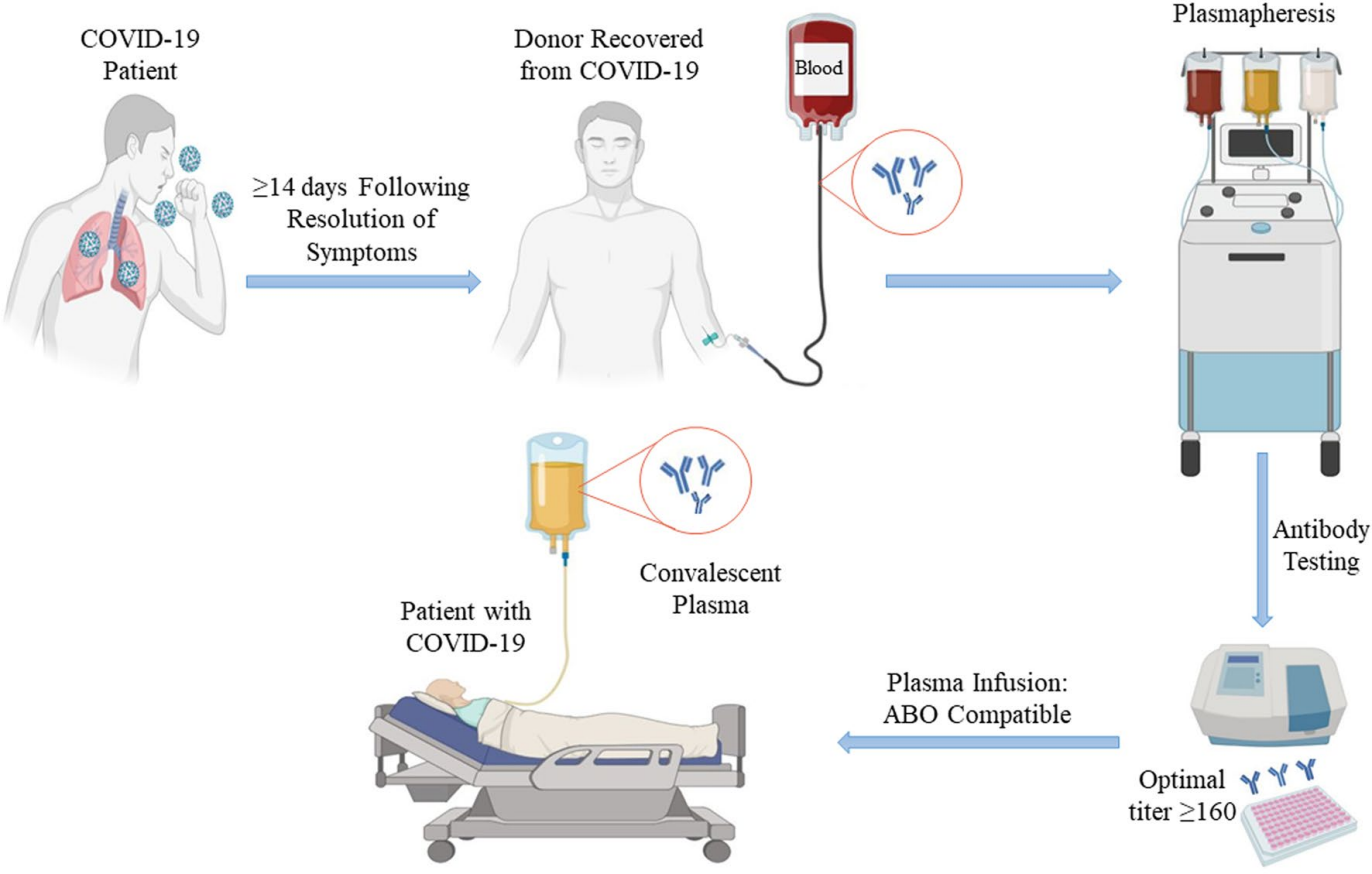
Schematic representation of the use of convalescent plasma to treat COVID-19 patients. The figure is made with biorender (https://biorender.com/)

Convalescent plasma (CP) therapy includes injection of plasma from recovered patients to people who are yet infected. CP can prevent the infection progression and reverse the inflammatory process through several mechanisms. The present neutralizing antibodies (NAbs), immune-modulatory cytokines and autoantibodies in convalescent plasma mediate its therapeutic effects. An antibody may bind to the virus, thereby directly neutralizing its infectivity, also other pathways mediated by antibody, like complement activation, phagocytosis and/or antibody-dependent cellular cytotoxicity can also contribute to its therapeutic effect [67, 68]. During apheresis, in addition to NAbs, other proteins such as natural antibodies, anti-inflammatory cytokines, clotting factors, and other undefined proteins are collected from donors [69]. In this sense, CP transfusion in infected patients can provide additional benefits, such as immunomodulation, by improving the severity of the inflammatory response. The stages of convalescent plasma therapy are illustrated in Fig. 1.

More than 1 year since the pandemic began, there are several reports of application of CP in the treatment of patients with SARS-CoV-2 infection. However, the benefit of this approach remains controversial. The results of a study to evaluate the effect of CP on mortality between patients with COVID-19 showed that patients with SARS-CoV-2 infection administered with CP (within 3 days of hospital admission) indicated a lower mortality rate compared with patients receiving standard care [70]. The data of another research demonstrated uncertain estimates on the effectiveness of CP for COVID-19 [71]. There is low certainty evidence of a probable decrease in mortality and mechanical ventilation, rapid viral clearance and the absence of any significant adverse events. On the other hand, CP has significant limitations such as lack of standard dosing methods, lack of availability to sufficient donors and host interactions and adverse effects, which can limit its usage [72, 73]. Indeed, for approval of CP as a potential treatment for COVID-19, designing extensive, good quality and adequately powered randomized clinical trials is needed.

#### Monoclonal antibody therapy

Monoclonal antibodies (mAbs), as a novel class of antibodies, are primarily developed by a specific group of B cells against a particular epitope of an antigen. mAbs therapy is a new era in the prevention of infectious disease, which can be derived from blood of the convalescent patients or engineered in the laboratory. This method can overcome many limitations in terms of specificity, safety, purity, and risk of blood-borne pathogen associated with plasma therapy and intravenous immunoglobulin preparations. The mAbs are involved in the treatment of infectious diseases by binding to a specific target. This binding is very diverse and can mimic, block or alter precise mechanisms to provide a very specific treatment for diseases with the appropriate therapeutic intervention [74].

##### Anti-host monoclonal antibodies

SARS-CoV-2 infections due to overproduction of inflammatory cytokines and chemokines, along with disruption of innate defense mechanisms, lead to a lack of immune response regulation also cause cytokine storms. For this reason, older people and people with certain diseases, such as patients with cardiovascular disease, diabetes, and cancer, may be prone to COVID-19 due to their lack of immunity and their tendency to exaggerated inflammatory responses [75]. Currently, significant efforts have been made to assess the effectiveness of different mAbs that regulate the immune system, and target the pathways of SARS-CoV-2 infection, to control the immune response and prevention of the cytokines storm in severe COVID-19 patients. In this context, monoclonal antibodies block inflammatory mediators, such as IL-1β, IL-2, IL-6/IL-6R, TLR3, IL-8, IL-12/IL-23, IL-17, TNFα, G-CSF, GM-CSF, ITAM (immune-receptor tyrosine-based activation motif), MCP1α, MIP1β, IP-10/CXCL10 (interferon γ-inducible protein 10), and complement component-5 [76–79].

Several studies reported the elevation of IL‑6 levels in patients with SARS-CoV-2 infection, which correlates with disease severity [23]. Between the excessive cytokines released by activated macrophages, IL-6 is one of the key cytokines. IL‑6 is released in response to acute inflammation, induction of TNF‑α, lipopolysaccharides, and IL‑1 [23]. The binding of IL-6 to the transmembrane or soluble form of the IL-6 receptor (IL-6R) stimulates the downstream gp130/JAK/STAT pathway [80]. Over-activation of IL-6 signaling causes numerous biological processes that help to organ damage. In many countries, the safety, efficacy, and pharmacodynamics of tocilizumab, as an IL-6 receptor-targeted mAb, have been evaluated to reduce the inflammatory response in severe COVID-19 patients. It is approved by the US FDA for the treatment of severe cytokine release syndrome during COVID-19 [81]. A meta-analysis of randomized clinical trials showed that tocilizumab was associated with a lower mortality rate in COVID-19 prospective studies [82]. The CDC panel recommends using tocilizumab (single IV dose of tocilizumab 8 mg/kg actual body weight up to 800 mg) in combination with dexamethasone [6 mg daily for up to 10 days] in certain hospitalized patients who are exhibiting rapid respiratory decompensation due to COVID-19.

IL-1β plays an important role in the cytokine storm associated with COVID-19 infection [76]. Anakinra is a recombinant, non-glycosylated human IL-1 receptor antagonist, which has a highly safe profile and large dosages have been utilized even in patients with severe viral illnesses, such as EBV, H1N1, and Ebola [83]. A recent cohort study investigated the anakinra effect on hyperinflammatory state, in SARS-CoV-2-infected patients. It was reported that anakinra significantly decreased both the requirement for invasive mechanical ventilation in the intensive care unit (ICU) and mortality between patients with severe SARS-CoV-2 infection, without substantial adverse effects. Up to this date, there are 38 ongoing clinical trials that investigate the use of anakinra as a promising treatment for SARS-CoV-2 infection [24]. A systematic review and patient-level meta-analysis demonstrated that anakinra could be a safe and anti-inflammatory medication to decrease the mortality rate in moderate to severe COVID-19 hospitalized patients, especially in the existence of signs of hyper inflammation, such as C-reactive protein concentrations higher than 100 mg/L [84]. In addition, another meta-analysis concluded that administration of anakinra in COVID-19 patients decreases both the requirement for mechanical ventilation and mortality rate of hospitalized non-intubated COVID-19 patients without higher serious adverse events [85]. However, more investigations are needed to the approval of anakinra as a potential treatment for COVID-19.

IL-17 operates ‘upstream’ of both IL-1 and IL-6, so it has been suggested that IL-17 can be involved in hyper-inflammatory syndrome in COVID-19. A phase 2 clinical trial (NCT04403243) on secukinumab, the IL-17-specific mAb, is currently underway. One trial showed that administration of netakimab as an anti-IL-17 mAb in patients with severe COVID-19 was related to clinically significant improvements, reduction of inflammatory biomarkers, and an improvement in oxygenation levels without significant adverse events [86]. However, this drug does not affect requiring mechanical ventilation and mortality.

GM-CSF (granulocyte–macrophage colony-stimulating factor), an important proinflammatory cytokine, plays an essential role in the immunopathogenesis of COVID-19 [87]. Hence, anti-GM-CSF therapy has been used to hospitalized severe COVID-19 patients, and clinical trials have indicated the efficacy of GM-CSF blockade for COVID-19 [87]. In the announced clinical trials, GM-CSF mAbs, including mavrilimumab, lenzilumab, and tocilizumab have been used in the treatment of SARS-CoV-2-infected patients [88]. A meta-analysis of GM-CSF antibody therapy for COVID-19 patients was performed, and a total of 12 qualified studies with 8979 [experimental group (*n* = 2673) and control group (*n* = 6306)] COVID-19 patients were included [88]. The results showed that the GM-CSF antibody therapy was associated with a 23% reduction in mortality rate. GM-CSF mAbs showed no effect on improving ventilation in COVID-19 patients and secondary infection. Overall, they concluded that GM-CSF antibodies can reduce respiratory symptoms and can be beneficial for severe COVID-19 patients [88]. However, the safety and efficacy of GM-CSF mAbs in the treatment of SARS-CoV-2-infected patients are controversial and more random clinical trials are required to evaluate these therapeutics in COVID-19.

A brief description of mAbs currently in development for COVID-19 is given in Table 2.

**Table 2:** Neutralizing monoclonal antibodies under investigation in clinical trials against COVID-19

Target	Name	Related disease	Trial ID
IL-6 receptor	Tocilizumab	Rheumatoid arthritis	NCT04730323
	Sarilumab	Rheumatoid arthritis	NCT04315298
	Levilimab	Rheumatoid arthritis	NCT04397562
IL-6	Siltuximab	Rheumatoid arthritis	NCT04329650
	Clazakizumab	Rheumatoid arthritis	NCT04494724
	Sirukumab	Rheumatoid arthritis	NCT04380961
	Olokizumab	Rheumatoid arthritis	NCT04380519
IL-1 receptor	Anakinra	Rheumatoid arthritis	NCT04366232
IL1 β	Canakinumab	systemic juvenile idiopathic arthritis and active Still's disease	NCT04362813
GM-CSF receptor	Mavrilimumab	Rheumatoid arthritis	NCT04447469
GM-CSF	Lenzilumab	Chronic myelomonocytic leukemia, juvenile myelomonocytic leukemia	NCT04583969
	Gimsilumab	Rheumatoid arthritis, multiple sclerosis, asthma, cancer	NCT04351243
	Otilimab	Rheumatoid arthritis	NCT04376684
	TJ003234	Rheumatoid arthritis, cytokine release syndrome, osteoarthritis	NCT04341116
IFN-γ	Emapalumab	Hemophagocytic lymphohistiocytosis	NCT04324021
TNF-α	Adalimumab	Rheumatoid arthritis	NCT04705844
	Infliximab	Autoimmune diseases	NCT04425538
IL-17	Secukinumab	Psoriasis, ankylosing spondylitis, psoriatic arthritis	NCT04403243
	Ixekizumab	Autoimmune diseases	NCT04724629
IL-12/IL-23	Risankizumab	Moderate to severe plaque psoriasis	NCT04583956

##### Anti-virus monoclonal antibodies

Currently, major research has focused on the exploring of mAbs that target the pathogen protein. The S protein presents on the surface of coronavirus and plays a vital role in virus entry and inducing host immune responses [89]. Prior studies have recognized a variety of effective mAbs to prevent the virus from entering host cells by targeting the SARS-CoV S protein. Depending on the site of binding, the antibodies that bind to the S protein can inhibit conformational change of the S protein and block membrane fusion or block its interaction with ACE2. Hence, the S protein could be considered as a key target to develop effective mAb against SARS-CoV-2. The critical target of mAbs is RBD (residues 318–510) of the S protein [90].

The FDA has currently issued emergency use authorizations for eight anti-SARS-CoV-2 monoclonal antibody products to treat mild to moderate COVID-19 in nonhospitalized patients with laboratory-confirmed SARS-CoV-2 infection who are at high risk of progressing to severe disease and/or hospitalization. These clinical use mAbs are include: bamlanivimab and etesevimab (from Eli Lilly); cilgavimab and tixagevimab (from AstraZeneca); casirivimab and Imdevimab (from Regeneron); regdanvimab (from Celltrion); and sotrovimab (from GSK and Vir Biotechnology) [91].

These therapeutic mAbs have indicated high effectiveness in trial studies with a decrease of 70–85% in hospitalization or death. Furthermore, 2B04 and 47D11 from AbbVie; BRII-196 and BRII-198 from Brii Biosciences; and TY027 from Tychan are under Phase III trials [91]. Given the emergence of different SARS-CoV-2 variants in various parts of the world, it was reported that RBD-specific NAbs are variably effective against these variants.

According to last update CDC’s COVID-19 guideline (September 29, 2021) (https://www.covid19treatmentguidelines.nih.gov/), in people with mild to moderate COVID-19 who are at high risk of progressing to severe disease and/or hospitalization, the combination of bamlanivimab and etesevimab has been found to provide a therapeutic benefit. The Delta variant, which has the L452R and T478K mutations, has demonstrated susceptibility to these mAbs combination in laboratory studies [92]. Because, in recent months, the Delta variant is the predominant variant circulating in all states of the United States, bamlanivimab (700 mg) plus etesevimab (1400 mg) to be administered as an IV infusion as one of the recommended treatment options for this patient population [93]. BLAZE-2, a Phase 3, randomized, placebo-controlled trial, is currently the only clinical trial that supports the use of either of these mAbs for post-exposure prophylaxis [94]. In laboratory studies, the Delta variant is also susceptible to casirivimab (600 mg) plus imdevimab (600 mg) and to sotrovimab (500 mg), so these products continue to also be recommended for this patient population [95]. Interestingly, newly known RBD core-binding NAbs SARS2-38 and LY-CoV1404 as monotherapy strongly neutralize all SARS-CoV-2 variants [91].

The cocktail of mAbs can exhibit more efficient anti-virus activity that could improve treatment efficacy and inhibit viral escape. Although this approach has promising results in infection neutralization, large-scale creation of monoclonal antibodies is costly, labor intensive, and time-consuming, especially against emerging pathogens. However, with recent advances in protein production platforms, antibody production can be made more cost-effective. It is possible to clone and express the sequences of effective monoclonal antibodies in proper expression systems, such as mammals, yeasts or plants, and then recombinant monoclonal antibodies could be tested [74]. Other challenges are determining at-risk individuals who benefit most from prophylactic neutralizing mAbs, optimum timing for administration of neutralizing mAbs, duration of protection provided by these mAbs and any potential effect of mAb therapy on subsequent vaccination [96].

#### Intravenous immunoglobulins therapy

Intravenous immunoglobulin (IVIG) contains the natural therapeutic immunoglobulin G (IgG), is isolated from the plasma of several thousand healthy donors. It is one of the immunotherapy methods used to treat various autoimmune and inflammatory diseases. In addition to clinically confirmed pathological conditions, IVIG has been evaluated as an unlabeled drug in more than 100 diseases. IVIG uses its therapeutic benefits in autoimmune diseases via several non-specific mechanisms that target mediators of the inflammatory immune response. The latest evidence from Shao et al. and other studies suggest that early initiation and high-dose injection of IVIG therapy may be helpful in the recovery of critical ill COVID-19 patients [97].

While the its mechanisms are still not exactly understood, the decline in inflammatory mediators following IVIG treatment indicate that IVIG may be effective in cytokine storming in hospitalized COVID-19 patients through scavenging of complement and inhibition of innate immune cell activation [98]. In a recently published paper, Cheng et al. found that SARS-CoV-2 encodes a superantigen-like motif near its S1/S2 cleavage site that is not present in other SARS family coronaviruses [99]. The authors conclude that this motif may explain the unique potential of SARS-CoV-2 in causing the cytokine storm observed in COVID-19. Since IVIG has antibodies that react against SARS-CoV-2 antigens, IVIG may suppress superantigen-mediated T cell activation and release of cytokine [99].

Up to date, 29 clinical trials are conducted in many countries to evaluate the impact of IVIG on patients with severe SARS-CoV-2 infection. However, the advantages of IVIG treatment for COVID-19 remains controversial. Recently, a meta-analysis was performed to assay the efficacy of IVIG therapy for COVID-19 patients [100]. In this meta-analysis, four clinical trials and three cohort studies containing 825 hospitalized patients are retrieved. Their results showed that the severity of COVID-19 is associated with the efficiency of IVIG. In the group of patients with critical conditions, IVIG could decrease mortality compared with the control group.

Alternatively, due to the broad-spectrum anti-inflammatory possessions of IVIG, this treatment can be combined with IL-1 and IL-6 targeted-immunotherapies, which show promising responses to explore therapeutic effects of additive or synergistic therapy [76, 78, 98].

Nevertheless, there is insufficient evidence for the approval of this therapeutic option and its beneficial effects for COVID-19 patients.

### Cell therapy for COVID-19 treatment

**Fig. 2:**
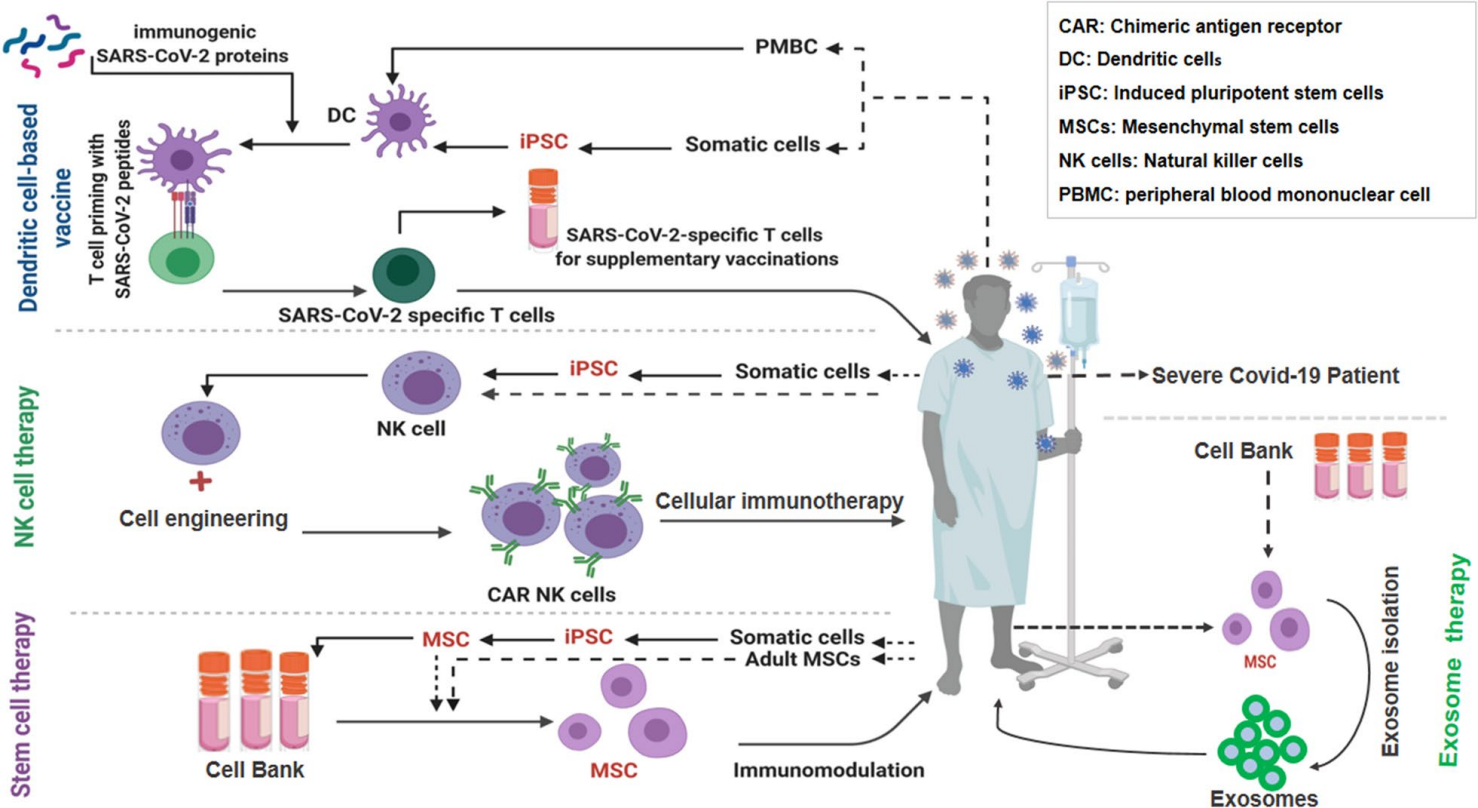
Summary schematic of immune cell-based therapy, which can be used to treat COVID-19 with permission from [128]. This figure represents a strategy to produce a cell-based products to prevent and treat COVID-19 patients. The figure is made with biorender (https://biorender.com/)

Currently, several cell-based therapy approaches including Mesenchymal stem cells (MSCs), Natural Killer (NK) cells, dendritic cells (DCs), engineered lymphocytes, new cell-based vaccine platforms, and Extracellular Vesicles are undergoing clinical trials to accept an approved therapeutic of COVID-19. These therapeutic approachs and their clinical application concepts for use in COVID-19 treatment are discussed below (Fig. 2).

#### Stem cell-based therapy

Soon after the COVID-19 outbreak, stem cell researchers suggested using MSCs, a suitable therapeutic candidate, to treat severe COVID-19 patients, and immediately its clinical trials were commenced. Due to migrating and homing ability, immunoregulatory and immunomodulatory function, anti-inflammatory effects, and stemness differentiation potential, it seems that MSCs could be demonstrated effects therapeutically in ARDS like severe and critical pneumonia COVID-19 [101, 102]. MSCs can be isolated from diverse adult tissues, including optionally bone marrow (BM), peripheral blood (PB) and adipose tissues (AT) (such as abdominal fat, buccal fat pad, and infrapatellar fat pad) and as well as neonatal birth-associated tissues, including placenta (PL), cord blood (CB) and umbilical cord (UC), Warton jelly (WJ), amniotic fluid (AF), and then stored for future desirable clinical applications [103–105]. It is hypothesized that MSCs could improve acute lung damage and repress the cell-mediated inflammatory response caused by SARS-CoV-2 [106]. Besides, MSCs are resistant to infection, and they lack the ACE2 receptor that SARS-CoV-2 applies for viral entry into cells [107, 108]. For COVID-19 treatment, MSCs can increase the lymphocyte count and regulatory DCs to boost their antiviral property, which results in the reduced level of C-reactive protein and pro-inflammatory cytokines (IL-6, TNFα, IL-8, etc.). These markers are the central markers of inflammation and ROS to diminish inflammation and oxidative stress. On the other hand, MSCs can increase the level of IL-10 as an anti-inflammatory protein activating regulatory cells, such as Tregs [102, 109]. Hence, MSCs play a central role in immune homeostasis by associating with cytokines, chemokines, and cell surface molecules [109]. Conclusively, all these immunomodulatory characteristics provide considerable potential for MSCs in clinical therapies.

During the COVID-19 pandemic, several clinical studies published clinical results on MSC-based therapy for COVID-19 patients with safety results and/or without serious unfavorable effects (Table 3) [101]. Umbilical cord tissues (especially umbilical cord blood and Wharton’s jelly), bone marrow, menstrual blood, and the elected MSC-derived products of companies are the primary MSC sources used in clinical trials. Remestemcel-L (Cellavita Mesoblast) and NestCell^®^ (Cellavita) are the two leading companies in introducing MSC therapy for use in COVID-19 clinical trials [101].

**Table 3:** List of published MSC-based clinical studies for COVID-19 treatment

Source of MSCs	Study type and number of enrollment (N)	Country	Results	References
Umbilical cord	Double-blind clinical trial phase 1/2a; 24	USA	No adverse events, decreasing IFN-γ, IL-6, TNF-α, and GM-CSF	[111]
Umbilical cord	Clinical trial phase 2; 101	China	Reducing the proportions of solid component lesion volume, no adverse events	[112]
Umbilical cord	Clinical trial phase 1;18	China	No adverse events, Decreasing IFN-γ, TNF-α, IL-6, and IL-1	[113]
Umbilical cord	Randomized clinical trial; 41	China	No adverse effects, decreasing CRP and IL-6	[114]
Umbilical cord and placental	Randomized clinical trial phase 1; 11	Iran	No serious adverse events, decreasing TNF-α, IFN-γ, IL-8, IL-6, and CRP	[115]
Menstrual blood-derived MSCs	Pilot clinical trial; 2	China	FiO21 decreased, SaO22 and PO23 improved, and adsorbing bilateral lung exudate lesions	[116]
Menstrual blood-derived MSCs	Phase I clinical trial; 44	China	Lower mortality, SpO2 was improved	[117]
AT-MSC	Randomized clinical trial phase 1; 13	Spain	No adverse events, reducing IL-6, CRP, LDH ferritin, and D-dimer, and increasing lymphocytes	[118]
Unknown	Pilot clinical trial; 10	China	No adverse events reduced CRP and TNF-α, increased IL-10, normalization of immune cell populations	[119]

Wang et al. performed a systematic review and meta-analysis to evaluate the safety and effectiveness of MSCs therapy for COVID-19-infected patients [110]. Twenty two studies containing 371 patients were included in their study. Allogeneic MSCs from different sources were injected in 247 participants. The results demonstrated that MSC therapy significantly decreased the occurrence of adverse events and mortality and the difference compared with the control group was statistically significant. No significant adverse events related to MSC therapy were observed. Pulmonary function, radiographic analysis, and inflammation- and immunity-associated biomarker levels all indicated improving trends. They concluded that MSC therapy is an effective and safe approach for the treatment of COVID-19-related pneumonia [110].

Nevertheless, there are no extensive data to estimate MSC’s role for the treatment of COVID-19, and no MSC-based therapies have been approved by the FDA in this regard. Hence, more research studies and clinical trial results are needed to clarify the precise mechanism by which MSCs support COVID-19-infected patients. Several clinical studies are ongoing in this regard.

MSC therapy may be an alternative approach for treating COVID-19, notably in patients with ARDS. However, several limitations have been identified in this respect. Main limitations including in failed up-scaling of the product to large-scale supply, cost issue, and the absence of translation to efficient clinical application (e.g., cell expansion from the starting material, cell banking, cell viability problems after thawing, and suboptimal delivery route), which could account for the restrictions of these studies.

#### Other cell-based therapies

The next candidate for cell-based therapy for COVID-19 patients is adoptive NK cells and CAR-NK cells. The NK cells are cytotoxic lymphocytes that include 10–15% of total peripheral blood white blood cells in humans. These cells have a critical role in linking during viral infection, the host cells shift to more responsive to NK cells and occur rules through: (I) upregulation of self-encoded molecules by infection or cellular stress; (II) downregulation of ligands for inhibitory receptors which suppress NK cell activation; and (III) direct identification of viral parts [120]. Recently, Novocellbio (Incheon, South Korea) reported promising results using NOVO-NK, an autologous NK cell treatment, in both conditions in vitro and in vivo. Hence, the company is leading further preclinical studies to analyze the mechanisms used by NOVO-NK therapy against SARS-CoV-2 [101]. Recently, a number of clinical trials have been conducted to investigate the safety and immunogenicity of intravenous infusion of peripheral blood mononuclear cells-derived NK cells from healthy donors in SARS-CoV-2-infected patients.

Moreover, an individual’s monocytic–dendritic cells are pulsed with SARS-CoV-2 peptides and used to prime the same individual’s T cells to produce SARS-CoV-2-specific immune cells. These lymphocytic cells may be cryopreserved or infused into the vulnerable individual as prevention or treatment against COVID-19 [101]. DCs related to immunotherapy and cell-based vaccination, are the next cell-based therapy candidate for the treatment of COVID-19. In the USA, an immuno-oncology company designing personalized vaccines has recently conducted a clinical trial (NCT04386252) of a vaccine of autologous DCs loaded with antigens from SARS-CoV-2, with or without GM-CSF, to inhibit COVID-19 in adults [121, 122]. These DCs consist of peripheral blood monocyte-derived DCs collected from the blood samples of patients. However, there is no more information about the results of this trial.

### Nutraceutical supplementation

Some clinicians recommend the intake of vitamin and mineral supplements to combat respiratory viral infections. Recently, adjunctive therapies are frequently used in the inhibition and/or treatment of SARS-CoV-2 infection or its complications. Vitamins C and D due to their immunomodulatory properties have received more attention to fighting against COVID-19 and it is estimated that these supplements may play a supportive role in infected patients and boost their immune system function [123].

An overview of systematic reviews of the association of vitamin D, vitamin C, zinc, and melatonin with inflammatory markers determined that using 50,000 IU/month of vitamin D leads to regulation in C-reactive protein (CRP), an inflammatory marker [124]. An intake of 1–2 g/day of vitamin C showed effectiveness in CRP and endothelial function, and a dose of 50 mg/day of zinc nutraceutical demonstrated favorable changes in CRP. An amount of melatonin ranging from 5 to 25 mg/day showed positive effects in CRP, TNF and IL-6 [124]. Hence, hospitalized SARS-CoV-2-infected patients indicated malnutrition and deficiencies in vitamin C, D, B12 selenium, iron, omega-3, and medium and long-chain fatty acids [125], these findings may be useful in the inhibition and treatment of COVID-19 patients. Some clinical trials were designed to evaluate some of these supplements. A pilot study of high-dose vitamin C in critically ill COVID-19 patients showed statistically improvements in oxygenation from baseline to day 7 in the treatment group compared to the control group [126]. A randomized clinical trial recommended the using 5000 IU daily vitamin D3, even for a short period, as an adjuvant therapy for COVID-19 patients with suboptimal vitamin D status [127]. The results of their study indicated that the time to recovery for cough and gustatory sensory loss between mild to moderate COVID-19 patients with suboptimal vitamin D status decrease with 2 week intake of 5000 IU vitamin D3 supplementation.

However, at present, there is inadequate data to recommend either for or against the intake of vitamins and mineral supplements for the inhibition or treatment of SARS-CoV-2-infected patients. More clinical trials are required to determine the complete role and implications of nutrition in the COVID-19 outbreak.

## Conclusion

The SARS-CoV-2 pandemic is a “public health emergency of international concern”. During this pandemic, many therapeutic strategies have been investigated for this deadly disease, including antivirals, anti-inflammatories, cell therapy, plasma therapy, monoclonal antibodies and intravenous immunoglobulin therapy. According to the results of several clinical studies, the drug repurposing strategy has confirmed its critical role in the rapid discovery of an efficient treatment option for COVID-19. Neutralizing mAb therapy is a promising and attractive approach for rapid prophylactic and treatment settings. Moreover, in conditions, where vaccines have less efficacy such as in immunocompromised people, young, elderly, and vaccine-hesitant individuals, mAb therapy may be an important option for SARS-CoV-2 infection. According to the findings of several clinical trials, cell therapy also improves COVID-19 patient recovery with safety and good immune tolerance. However, at present, there is no specific and approved option for treating SARS-CoV-2 infection yet and more studies are needed to determine the safety and efficacy of current treatment strategies. Although the COVID19 pandemic has brought great challenges, the continued efforts to combat the virus and the excellence and hard work of many researchers around the world give us hope of winning this battle in the near future.

## Data Availability

Not applicable.
